# Stocking density mediated stress modulates growth attributes in cage reared *Labeo rohita* (Hamilton) using multifarious biomarker approach

**DOI:** 10.1038/s41598-022-13570-x

**Published:** 2022-06-14

**Authors:** Himanshu Sekhar Swain, Basanta Kumar Das, Aurobinda Upadhyay, Mitesh Hiradas Ramteke, Vikas Kumar, Dharmendra Kumar Meena, Uttam Kumar Sarkar, Narinder Kumar Chadha, Kiran Dube Rawat

**Affiliations:** 1 ICAR-Central Inland Fisheries Research Institute, Barrackpore, Kolkata, 700 120 India; 2 ICAR-Central Institute of Fisheries Education, Yari Road, Versova, Mumbai, 400 061 India

**Keywords:** Biochemistry, Biological techniques, Ecology, Environmental sciences

## Abstract

The present study was conducted for 240 days to evaluate the effects of stocking density based on growth attributes, digestive enzymes, muscular composition, biochemical and physiological responses of *Labeo rohita* fingerlings in tropical inland open water cages. *L. rohita* (30.35 ± 1.08 g) were randomly distributed into three treatments, namely low stocking density, LSD (10 m^−3^), medium stocking density, MSD (20 m^−3^) and high stocking density, HSD (30 m^−3^) in triplicates. Fish were fed twice daily with CIFRI CAGEGROW® floating feed (crude protein-28%, crude fat-4%). Fish growth and feed efficiency were higher (*p* < 0.05) in LSD, however, MSD registered a higher yield. Amylase and protease activity reduced whereas lipase activity increased with increasing stocking density. Muscle crude protein and crude fat formed an inverse correlation. The fillet quality deteriorated at higher stocking densities based on Muscle pH, drip loss and frozen leakage rate. The stress biomarkers level (glucose, cortisol, superoxide dismutase and catalase) increased in serum under crowding conditions. Glutamate oxaloacetate transaminase and glutamate pyruvate transaminase in serum were significantly increased in HSD. Serum protein levels decreased with the increase in stocking densities. Body ionic imbalance (Na^+^, Cl^−^ and K^+^) was observed under crowding stress. Based on growth attributes and multiple biomarker responses, *L. rohita* @ 10 m^−3^ was found to be the optimum density for inland open water cage culture.

## Introduction

Stocking density optimization is a prerequisite for the development of the protocol for practice of any candidate fish species and it differs based on species and its life stages, types of production system and the management practice followed^1^. Overstocking produces significant stress which leads to growth retardation, poor health, lower survivability and yield loss^2,3^. The stocking density and growth are inversely correlated owing to the concurrence for food and space which in turn elucidate stress in fish^4^. However, understocking leads to poor production due to the underutilization of available space and resources^2,5^. As the cage culture is an intensive farming system, the apparent efficiency of culture systems can be maximized by increasing the densities to optimal levels^6,7^.

Stressor in aquaculture covers a broad and diverse range of biotic^7^ and abiotic^8^ factors. Stocking density mediated stress has known physiological and behavioural consequences for the cultured fish^9^. The biochemical responses of fish to menacing stimuli are regularly controlled by physiological modification of the nervous and humoral system in order to maintain body homeostasis^10^. Fish subjected to stressors can drop the muscular flesh quality^11^, decreased digestive enzymes^12^ and altered the serum biochemical parameters^13^. Stress can also increase adrenaline which raise blood pressure resulting increase in the blood flow through the gill lamellae and altering the exchange of ions^14^.

Cage culture in inland open water is considered a relatively recent aquaculture innovation, has rapidly expanded during the past decades in fresh, estuarine, and marine open water bodies. Due to pressures for aquatic products across the globe, cage culture is presently undergoing swift changes^15,16^. In India, reservoirs and wetlands are the untapped open water resources suitable for freshwater cage culture^17^. India is bestowed with 19,370 reservoirs covering 15 states with an estimated area of 3.51 million ha surface area at its full capacity^18^. Utilizing a modest fraction of their surface area, cage culture can accord a notable quantity of fish production to the total fish production basket of the country. At present, *Pangasianodon hypophthalmus* occupies the centre stage in inland cage culture in India. However, problems and prospects of this species in cage culture cannot be neglected. Farmed *P. hypophthalmus* from India fetches low prices in the domestic market and export as well^19^. Hence diversification of species in inland cages in India, which is having good market demand, is the need of the hour^18^.

Indian major carp is the mainstay of the Indian aquaculture contributing 80% of the total country aquaculture production^20^, of which rohu (*Labeo rohita*) is in greater preference by the consumers. Despite a huge prospective for species diversification, the culture of *L. rohita* has been only restricted in ponds and tanks and no scientific information is available on its performances in cage culture. With this background, the present study was undertaken to bring out the optimum stocking density of *L. rohita* based on its growth attributes and by examining a variety of endpoints to measure its physio-biochemical responses in inland open water cage culture. This is the first report on grow out cage culture of *L. rohita* in reservoirs. The study will be extremely beneficial for aquaculturists and researchers for standardizing the cage culture protocol of *L. rohita* in tropical reservoirs of India and Southeast Asian countries.

## Result

### Growth performance and feed efficiency

The growth characteristics among the groups significantly differed (*p* < 0.05) (Table 1). Higher growth performances in terms of final body weight (FBW), weight gain (WG) and absolute growth rate (AGR) were recorded in lower stocking density (*p* < 0.05). The value of specific growth rate (SGR) was (1.29 ± 0.01%) in LSD followed by MSD (1.21 ± 0.01%) and HSD (1.07 ± 0.03%). An increase in stocking density led to a decline in fish survival percentage (*p* < 0.05) from LSD (88 ± 0.11%) to HSD (66 ± 0.18%). Fish reared at LSD reported higher (*p* < 0.05) feed and protein utilization (protein efficiency ratio—PER and feed conversion efficiency—FCE) than MSD and HSD. On contrary, the lowest feed conversion ratio (FCR) (1.96 ± 0.01) was displayed in LSD while medium and high density showed significantly deleterious FCR values (*p* < 0.05) (Table 1).

**Table 1:** Growth performance and feed utilization efficiency of *L. rohita* stocked at three different stocking densities.

Treatment	IBW (g)	FBW (g)	WG (g)	SGR (%)	AGR (g)	Survival (%)	FCR	PER	FCE	Yield (kgm^−3^)
LSD	30.35 ± 1.08	680.69 ± 21.30^a^	650.34 ± 21.30^a^	1.29 ± 0.01^a^	2.70 ± 0.08^a^	88.00 ± 0.11^a^	1.96 ± 0.01^c^	1.69 ± 0.01^a^	0.50 ± 0.02^a^	5.98 ± 0.53^b^
MSD	30.35 ± 1.08	560.52 ± 25.98^b^	530.17 ± 25.98^b^	1.21 ± 0.01^a^	2.20 ± 0.10^b^	72.00 ± 0.14^b^	2.56 ± 0.02^b^	1.29 ± 0.01^b^	0.38 ± 0.02^b^	8.059 ± 0.71^a^
HSD	30.35 ± 1.08	405.45 ± 32.79^c^	375.1 ± 32.79^c^	1.07 ± 0.03^b^	1.56 ± 0.13^c^	66.00 ± 0.18^c^	2.87 ± 0.01^a^	1.16 ± 0.02^c^	0.34 ± 0.03^c^	8.050 ± 1.13^a^

Values are mean ± SE. Means in the same column within each classification bearing different superscripts (a, b, c) are significantly different (*P* < 0.05).

*IBW* initial body weight, *FBW* final body weight, *WG* weight gain, *SGR* specific growth rate, *AGR* absolute growth rate, *FCR* feed conversion ratio, *PER* protein efficiency ratio, *FCE* feed conversion efficiency, *LSD* low stocking density, *MSD* medium stocking density, *HSD* high stocking density.

### Serum biochemical indices

**Figure 1:**
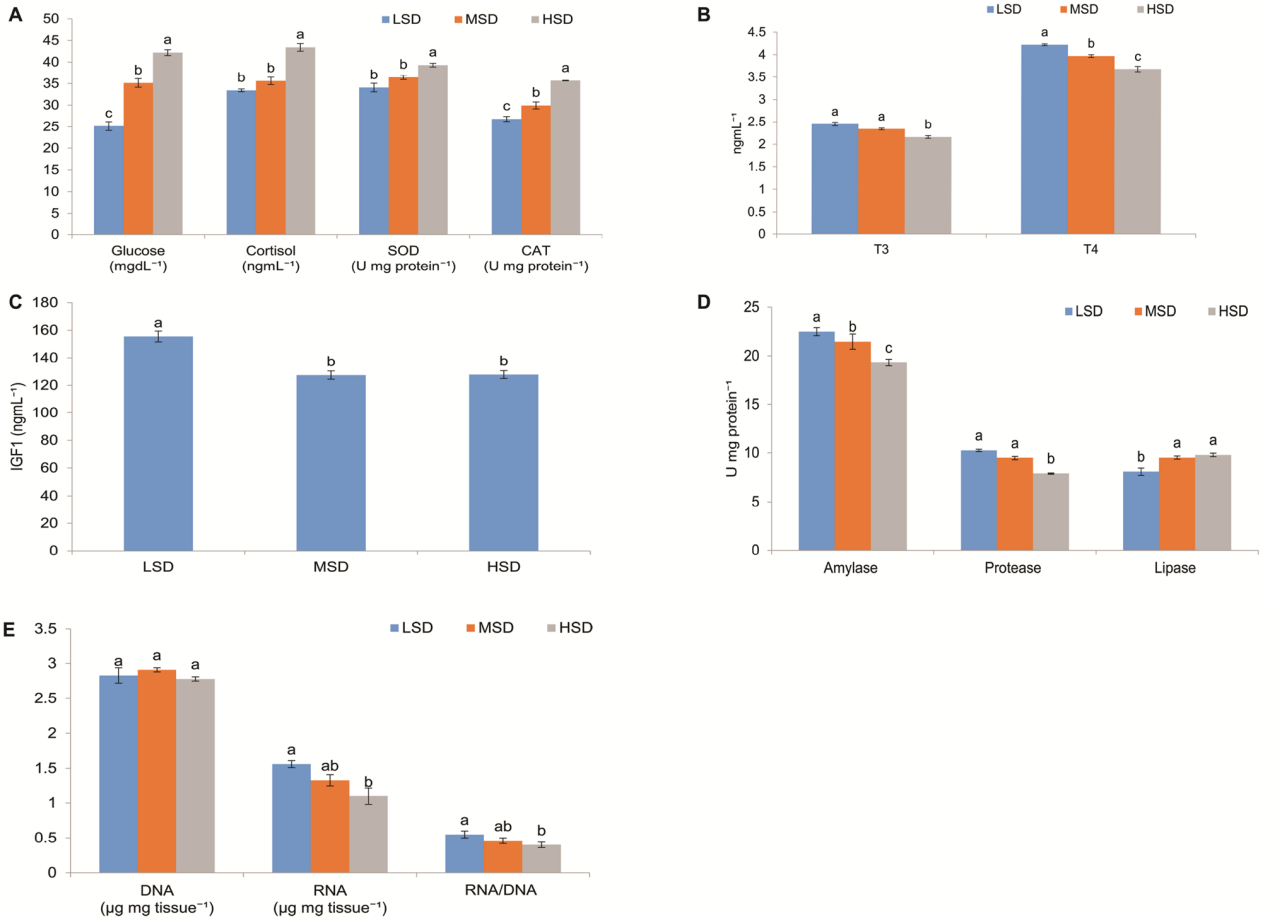
Activities of (**A**) glucose, cortisol, superoxide dismutase (SOD), catalase (CAT) in serum (**B**) triiodothyronine (T3) and thyroxine (T4) in serum, (**C**) insulin like growth factor 1 (IGF1) in serum, (**D**) amylase, protease and lipase in the gut, and (**E**) concentration of DNA, RNA and RNA/DNA ratio in the muscle of *L. rohita* stocked at three different stocking densities. Each bar represents values as mean ± SE. Within endpoints and groups, bars with different superscripts (a, b, c) are significantly different (*P* < 0.05).

Post hoc analysis signifies that the level of serum glucose markedly increased (*p* < 0.05) with an increase in stocking density (Fig. 1A). The glucose concentration was ranged from 25.14 ± 1.02 to 42.19 ± 0.68 mgdL^−1^ from LSD to HSD. The superoxide dismutase (SOD) level was increased significantly (*p* < 0.05) in HSD (39.31 ± 0.43 U mg protein^−1^) and the extent of increase in SOD was 15% from LSD (Fig. 1A). There were no significant (*p* > 0.05) changes in SOD levels among lower and medium densities. The catalase (CAT) value was significantly (*p* < 0.05) increased in MSD (29.93 ± 0.81 U mg protein^−1^) and HSD (35.69 ± 0.06 U mg protein^−1^) than LSD (26.80 ± 0.58 U mg protein^−1^) (Fig. 1A). Cortisol level demonstrated apparently higher in HSD with a factor of 29.89%, in comparison to LSD (*p* < 0.05). However, cortisol levels did not vary among MSD and HSD (Fig. 1A).

The concentration of serum glutamate oxaloacetate transaminase (SGOT) and serum glutamate pyruvate transaminase (SGPT) in LSD and MSD varied significantly from HSD (*p* < 0.05) as presented in Table 2, however, the level did not vary among low and medium density. The sum of total serum protein decreased significantly (*p* < 0.05) with increasing stocking density from 3.29 ± 0.01 gdL^−1^ in LSD to 2.33 ± 0.08 gdL^−1^ in HSD (Table 2). Albumin, which is an important carrier for various hormones like thyroid, steroids and fatty acids varied from 0.96 ± 0.01 to 0.72 ± 0.01 gdL^−1^ from lower to higher stocking density. Serum globulins responsible for blood clotting and immunological functions proclaimed a decreasing trend from lower to higher stocking density. The ratio of albumin and globulin was found to be non-significant (*p* > 0.05) amongst LSD and MSD but significant with HSD (Table 2).

**Table 2:** Serum biochemical indices of *L. rohita* stocked at three different stocking densities.

Treatments	SGOT (UL^−1^)	SGPT (UL^−1^)	Protein (gdL^−1^)	Albumin (gdL^−1^)	Globulin (gdL^−1^)	A:G ratio	Na^+^ (mmolL^−1^)	K^+^ (mmolL^−1^)	Cl^−^ (mmolL^−1^)
LSD	58.16 ± 1.72^b^	3.88 ± 0.04^b^	3.29 ± 0.01^a^	0.96 ± 0.01^a^	2.33 ± 0.01^a^	0.41 ± 0.01^a^	131.13 ± 0.54^a^	2.52 ± 0.02^a^	112.50 ± 4.04^a^
MSD	60.01 ± 2.89^b^	4.00 ± 0.04^b^	2.96 ± 0.05^b^	0.85 ± 0.01^b^	2.11 ± 0.04^b^	0.40 ± 0.01^a^	129.50 ± 0.28^b^	2.58 ± 0.07^a^	102.40 ± 3.41^ab^
HSD	66.09 ± 1.90^a^	4.51 ± 0.08^a^	2.33 ± 0.08^c^	0.72 ± 0.01^c^	1.61 ± 0.06^c^	0.44 ± 0.01^b^	130.50 ± 0.28^ab^	2.12 ± 0.05^b^	98.16 ± 1.30^b^

Values are mean ± SE. Means in the same column within each classification bearing different superscripts (a, b, c) are significantly different (*P* < 0.05).

*SGOT* serum glutamate oxaloacetate transaminase, *SGPT* serum glutamate pyruvate transaminase, *A:G ratio* albumin:globulin ratio, *LSD* low stocking density, *MSD* medium stocking density, *HSD* high stocking density.

The serum electrolytes like Na^+^, K^+^ and Cl^−^ were monitored and their values were represented in Table 2. The level of Na^+^, altered among lower and higher density treatments. Chloride ions decreased with the increased stocking density. The significant difference of K^+^ and Cl^−^ values (*p* < 0.05) have been observed in the lower and higher stocking density.

The serum thyroid level was estimated using the parameters T3 (triiodothyronine) and T4 (thyroxine) (Fig. 1B). The T3 value did not differ (*p* > 0.05) significantly except in HSD (2.16 ± 0.03 ngmL^−1^), however, T4 was decreased (*p* < 0.05) with an increase in stocking density and the lowest value recorded in HSD (3.68 ± 0.06 ngmL^−1^). The IGF1 (insulin like growth factor 1) value was highest at LSD (155.52 ± 3.92 ngmL^−1^) and it was significantly (*p* < 0.05) declined in both MSD and HSD (Fig. 1C).

### Digestive enzymes

The gut amylase, protease and lipase activities were analyzed and shown in Fig. 1D. Amylase was found to be inversely proportional to the stocking density and the value ranged from 19.30 ± 0.35 to 22.48 ± 0.41 U mg protein^−1^ from HSD to LSD. Protease, which is responsible for the breakdown of proteins into smaller polypeptides or single amino acids did not differ (p > 0.05) among LSD (10.25 ± 0.12 U mg protein^−1^) and MSD (9.46 ± 0.18 U mg protein^−1^) however it markedly differed in HSD (7.88 ± 0.05 U mg protein^−1^). The lipase activity was found to be indifferent among MSD and HSD but varied in LSD (*p* < 0.05).

### Chemical composition of muscle and flesh quality

The muscular chemical compositions (pH, drip loss—DL, frozen leakage rate—FLR) at different stocking densities were measured and presented in Table 3. The crude protein content showed significant variation between HSD and LSD. The decreasing trend of crude fat content among the treatments was observed though the values were insignificant between LSD and MSD. Stocking density had no impact (*p* > 0.05) on moisture and ash content. The muscular pH declined with an increase in stocking densities where the LSD showed the highest pH value (6.50 ± 0.11) and lowest at HSD (5.8 ± 0.17) as shown in Table 3. Muscular DL and FLR increased with increasing stocking density and the values significantly differed between LSD and HSD (Table 3).

**Table 3:** Muscle chemical composition and flesh quality of *L. rohita* stocked at three different stocking densities.

Treatments	Moisture (%)	Crude protein (%)	Crude fat (%)	Ash (%)	Muscle pH	Drip loss (%)	FLR (%)
LSD	77.67 ± 0.74^a^	13.82 ± 0.11^b^	2.03 ± 0.05^a^	3.72 ± 0.01^a^	6.5 ± 0.11^a^	2.1 ± 0.17^b^	0.7 ± 0.05^b^
MSD	77.03 ± 0.26^a^	14.45 ± 0.52^ab^	1.90 ± 0.06^a^	3.74 ± 0.01^a^	6.0 ± 0.05^b^	3.2 ± 0.11^a^	1.1 ± 0.11^ab^
HSD	75.61 ± 1.62^a^	15.28 ± 0.22^a^	1.63 ± 0.06^b^	3.72 ± 0.02^a^	5.8 ± 0.17^b^	3.8 ± 0.40^a^	1.4 ± 0.17^a^

Values are mean ± SE. Means in the same column within each classification bearing different superscripts (a, b, c) are significantly different (*P* < 0.05).

*FLR* frozen leakage rate, *LSD* low stocking density, *MSD* medium stocking density, *HSD* high stocking density.

### Nucleotide ratio

The ratio of RNA:DNA is an important sign of stress in the fishes as shown in the Fig. 1E. The quantity of DNA did not vary significantly (*p* > 0.05), however, the quantity of RNA varied significantly (*p* < 0.05) among LSD (1.33 ± 0.08 µg mg tissue^−1^) and HSD (1.56 ± 0.12 µg mg tissue^−1^). The nucleotide ratio (RNA:DNA) was found to be decreased with increasing stocking density (*p* < 0.05).

### Water quality parameters

The water quality parameters of the cage are depicted in Table 4. The range of mean water temperature, transparency, DO, pH and specific conductivity was 28.50–29.00 °C, 118–136 cm, 6.98–7.70 mgl^−1^, 7.50–7.85 and 79.50–87.21 mmhocm^−1^ respectively. The alkalinity, hardness, total nitrogen and total phosphorus ranged from 56.10 to 58.50 mg l^−1^, 48.16–56.00 mg l^−1^, 0.57–0.62 mg l^−1^ and 0.16–0.18 mg l^−1^ respectively. No significant variation was observed in water quality parameters among different treatments (*p* > 0.05).

**Table 4:** Variations in cage water quality parameters in cages.

Parameters	LSD	MSD	HSD
Water temperature (°C)	28.5 ± 1.20	28.50 ± 1.30	29.0 ± 1.20
Water transparency (cm)	118.00 ± 4.02	127.00 ± 5.12	136.00 ± 4.65
Dissolved oxygen (mgl^−1^)	7.20 ± 0.26	7.70 ± 0.31	6.98 ± 0.18
pH	7.85 ± 0.88	7.50 ± 0.90	7.70 ± 0.64
Specific conductivity (mmhocm^−1^)	87.21 ± 2.30	79.50 ± 3.50	82.20 ± 2.80
Alkalinity (mg l^−1^)	58.50 ± 0.56	56.10 ± 1.50	58.33 ± 1.02
Hardness (mgl^−1^)	49.52 ± 2.13	48.16 ± 1.28	56.00 ± 1.36
Total nitrogen (mgl^−1^)	0.60 ± 0.05	0.62 ± 0.04	0.57 ± 0.02
Total phosphorous (mgl^−1^)	0.16 ± 0.01	0.17 ± 0.04	0.18 ± 0.02

Values are mean ± SE. No significant variation was observed in water quality parameters among different treatments (*P* > 0.05).

*LSD* low stocking density, *MSD* medium stocking density, *HSD* high stocking density.

### Univariate and multivariate statistical analysis

**Figure 2:**
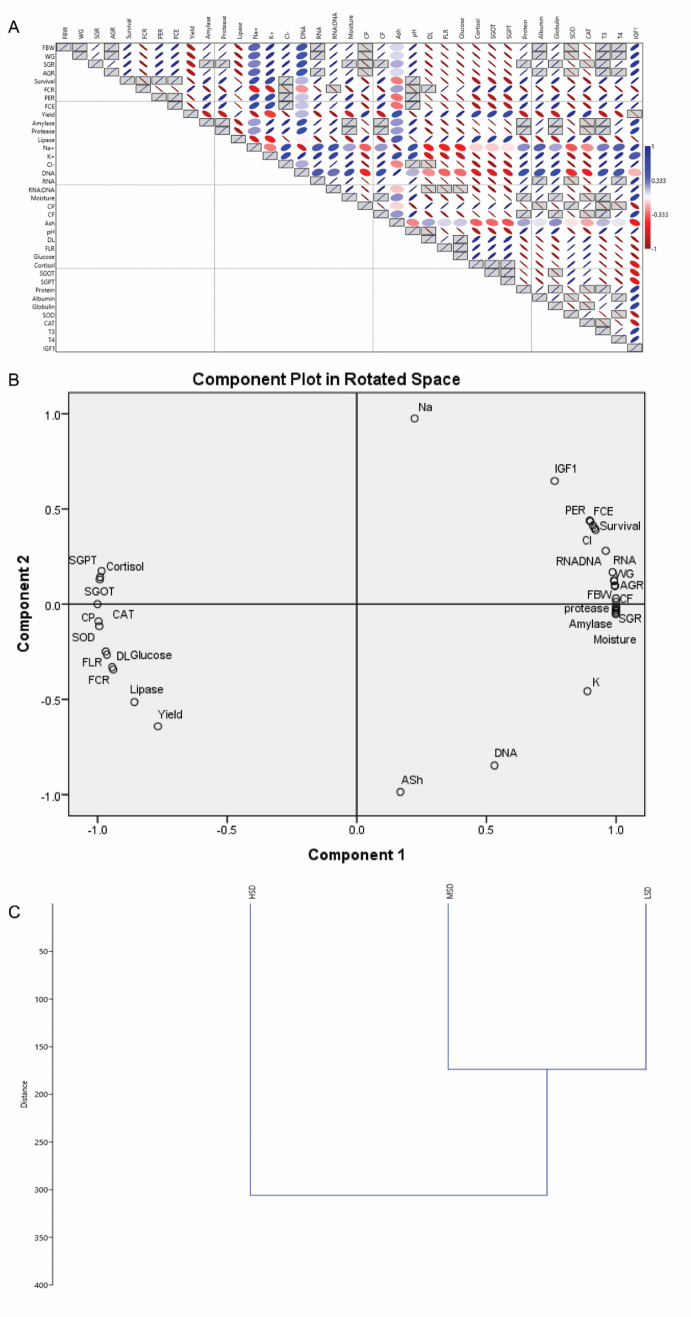
(**A**) *Pearson correlation coefficient, (**B**) **Principal component analysis, and (**C**) ***Hierarchical cluster analysis using growth attributes and multiple biomarkers of *L. rohita* cultured in three stocking densities in cages. *Red coloured small eclipse showed negatively correlated variables while blue coloured small eclipse shape showed positively correlated variables, large eclipse showed less correlated variables, boxed variables showing significantly (*P* < 0.05) correlated. **Extraction method: Principal component analysis, Rotation method: Varimax with Kaiser Normalization, ***Wards method with Euclidean method.

Pearson correlations among the parameters are depicted in Fig. 2A. The correlation coefficient suggested that correlated parameters are closely associated and their behaviours are interdependent to each other. This correlation was further confirmed by principal component analysis (PCA). Two PC were obtained having eigenvalues > 1. PC1 and PC2 constitute eigenvalues 32.23 and 4.76 and the variance 87.12% and 12.88% respectively. PC1 showed a strong positive correlation among FBW, WG, SGR, AGR, survival, PER, FCE, amylase, protease, K^+^, Cl^-^, RNA, RNA:DNA, moisture, CF, pH, serum protein, albumin, globulin, T3, T4, and IGF1 (Fig. 2B). However, a negative correlation exists among FCR, lipase, CP, DL, FLR, serum glucose, cortisol, SGOT, SGPT, SOD and CAT (Fig. 2B). PC2 didn’t show any significant correlation among the vital parameters. Hierarchical clustering dendrogram formed two groups among the three stocking densities. Cluster one was noticed between LSD and MSD while HSD formed a separate cluster (Fig. 2C).

**Figure 3:**
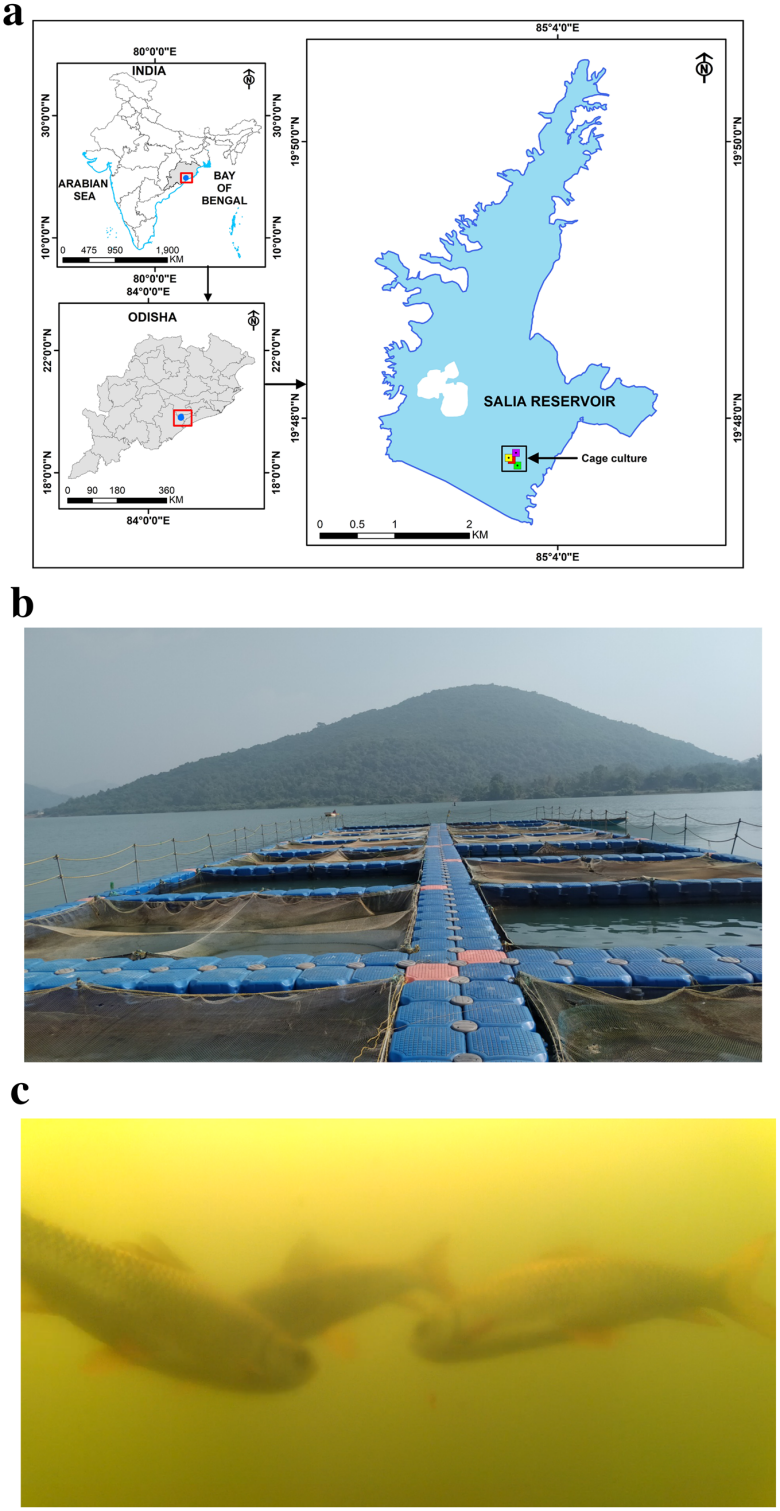
(**a**) GIS map* of experimental site, Salia reservoir, Ganjam district, Odisha, India (N19° 48.3887ʹ and E85° 03.6983ʹ). The reservoir area is 947.8 ha at its full reservoir level. The present experiment was conducted in 9 cages out of 58 installed in the reservoir. *GIS map was prepared by using ArcGIS software, version 9.3 (https://arcgis.software.informer.com/9.3/). (**b**) Experimental cage (HDPE) site, Salia reservoir, Ganjam district, Odisha, India. The photography was taken by the author (Dr. Himanshu Sekhar Swain). (**c**) Underwater image of *L. rohita* in the cage. The images were taken by using SONY-RX 100 VI underwater camera in the daytime during 10 AM to 2 PM. The underwater photography was undertaken by the scuba divers as well as by the authors with all safety features as per requirement and visibility in the water.

## Discussion

Stocking density, nutrition and appropriate culture environment are considered as vital factor which affects fish growth and production^21^ however overcrowding negatively affect the growth of fish^22^. In the present study, stress led by crowding has an obvious effect on growth attributes like FBW, WG, SGR, AGR and survival which are significantly different among LSD, MSD and HSD. The present findings proclaimed that the feed utilization and protein efficiency ratio was found significantly greater in lower stocking density. However, at higher stocking density, FCR, FCE and PER was deteriorated. The degradation in growth, biomass production and feed utilization in HSD could be the result of crowding, which triggered a rising demand for energy to activate the physiological functions to combat the stress by decreasing appetite and food intake and led to a reduction in the available energy for growth^23–25^. The major studies on evaluation of stocking density were observed on various tropical as well as temperate fishes and also in different culture systems. A similar effect of crowding stress on growth and feed utilization was also observed in common carp, *Cyprinus carpio* cultured in biofloc for 49 days^26^, in tanks for 60 days^27^ and glass aquaria for 30 days^28^; in grass carp, *Ctenopharyngodon idella* cultured in the tank for 60 days^29^ and tanks for 78 days^30^; in olive barb, *Puntius sarana* reared in cages for 90 days^7^; in Amazon fish, *Colossoma macropomum* cultured in net cages for 60 days^31^ and Nile tilapia, *Oreochromis niloticus* cultured in cages for 4 months^32^ and tanks for 84 days^33^. On contrary, many authors could not establish any relation between stocking density and growth attributes in silver perch, *Bidyanus bidyanus* cultured in cages for 210 days^34^ and Atlantic sturgeon, *Acipenser oxyrinchus* cultured in circular tanks for 26 days^35^. Thus, stocking density (10 m^−3^) of *L. rohita* ensured the best growth performances in terms of weight gain, survival and feed and protein utilization.

Glucose is considered an indicator of secondary stress response in fishes^36,37^. Stressors such as transportation, stocking density, confinement and bad handling are responsible for the increase in blood-glucose and whole body-glucose levels. The increased level of catecholamine results in the activation of glycogenolysis and gluconeogenesis, which ultimately leads to the rise of glucose levels in blood^38^. It is a secondary stress response that shifts the glucose to body tissue to cope with the restoration of energy-demanding activity. In the present study, increased blood glucose level (*p* < 0.05) from LSD to HSD was observed with an increment in stocking density followed by crowding stress. Similar findings were also observed in olive barb, *P. sarana* cultured in cages for 90 days^7^; in Gilthead seabream, *Sparus auratus* cultured in tanks for 116 days^39^; in Senegalese sole, *Solea senegalensis* cultured in partial-recirculated seawater system for 18 days^40^; in Chinese sturgeon, *Acipenser sinensis* reared in recirculating aquaculture system for 3 months^41^; in common carp, *Cyprinus carpio* cultured in biofloc for 49 days^26^, in tanks for 60 days^27^ and Asian seabass, *Lates calcarifer* cultured in circular cages for 6 months^42^, where stocking density mediated stress elevated the blood glucose level.

Free radicals and reactive oxygen species (ROS) are produced continuously under stressful conditions to combat the damage of antioxidant abilities and also act as a scavenger of excessive superoxide generated in the body. SOD and CAT played a key antioxidant enzyme in the animal defence system, to function against oxidative stress, and with the increase in stress; its value tends to ascend^43,44^. SOD level in serum of *L. rohita* at higher density increased significantly. Similar to the present findings, an increment of SOD and CAT with increased stocking density was observed in olive barb, *P. sarana* cultured in cages for 90 days^7^; in common carp, *Cyprinus carpio* cultured in biofloc for 49 days^26^ and tanks for 60 days^27^; in Nile tilapia, *O. niloticus* cultured in biofloc for 120 days^12^; in GIFT strain of Tilapia cultured in biofloc for 90 days^45^, and dourado, *Salminus brasiliensis* reared in the recirculating system for 80 days^10^.

Cortisol is released as an indicator of chronic and acute stress which elevates the expenditure of energy and hence the stored somatic energy expenses also subsequently increase, which ultimately led to growth retardation in fish^26,42^. The cortisol level of *L. rohita* elevated at high density (*p* < 0.05). Moreover, an increase in the level of cortisol and glucose were associated with the reduction in muscular fat^11^. In concordance with the present study, growth retardation and cortisol level positively correlated in olive barb, *P. sarana* cultured in cages for 90 days^7^; in common carp, *Cyprinus carpio* cultured in biofloc for 49 days^26^, in tanks for 60 days^27^, in glass aquaria for 30 days^28^ and tanks for 87 h of crowding^46^ and GIFT strain of Tilapia *O. niloticus* cultured in biofloc for 90 days^45^. In contrast to the present finding, cortisol level remained unaffected in African catfish, *Clarias gariepinus* cultured in tanks for 60 days^47^ at higher density but decreased in Nile tilapia, *O. niloticus* in biofloc based systems cultured for 120 days^12^.

The major thyroid hormones, triiodothyronine (T3) and thyroxine (T4) play an important role in the growth and development of fishes^48^ which are majorly affected by crowding stress, hypoxic condition and starvation^49^. The crowding stress plays a major role in the elevation of cortisol level and the increase in cortisol level has a negative feedback mechanism on the hypothalamus-pituitary-interrenal axis thus decreasing the peripheral circulation of T3 and T4. Unless the thyroid hormones are not in free form, they cannot be utilized by the fish/animal, leading to poor growth^50^. The elevated crowding stress led the fishes towards growth retardation, also towards less availability of thyroid hormone in fish serum, which was observed in the present study. In our study, the decrease in the level of T3 and T4 with an increase in stocking density was supported by the earlier findings in Amur sturgeon (*Acipenser schrenckii*) reared in concrete tanks for 70 days^51^; in mosquitofish (*Gambusia holbrooki*) cultured in glass aquaria for 8 weeks^52^; in channel catfish (*Ictalurus punctatus*) cultured in recirculating aquaculture system for 60 days^53^ and olive barb (*P. sarana*) cultured in cages for 90 days^7^.

The IGF1 axis has a prominent role in the regulation of various physiological responses in fishes^54,55^. Due to the secretion of IGF1, growth hormone has a prominent impact on growth regulation in fishes^56^. Due to the stress factors like overcrowding in the present study, the somatic growth was inhibited significantly in MSD and HSD; also, these groups of fishes were encountered with significantly lower IGF1 concentration in their serum. The findings were consistent with earlier studies where stress affects the secretion of IGF in tilapia, *Oreochromis mossambicus* stocked in circular tanks for 48 hours^57^; Chinese sturgeon, *A. sinensis* cultured in recirculating aquaculture system for 3 months^41^; Amur sturgeon, *A. schrenckii* cultured in concrete ponds for 70 days^58^; Senegalese sole, *S. senegalensis* cultured in fibreglass tanks for 60 days^59^. The result divulged that high cortisol levels under stressful conditions, inhibition the secretion of growth hormone and IGF1^60^.

SGOT and SGPT, both are ubiquitous aminotransferases in the mitochondrion of fish and are used as an indicator of hepatic ruination ^61^. The enzymes are coerced to be released into the bloodstream when the liver gets damaged. In the present finding, SGOT and SGPT activity upraised in *L. rohita* due to crowding stress in HSD; however, the change in such values among LSD and MSD was not varied significantly. This result might also reflect that increase of these enzymes in the serum was occurred due to the overutilization of hydrocarbons in order to meet the stress-mediated energy demands by fish. Similar types of outcome also observed in olive barb, *P. sarana* cultured in cages for 90 days^7^; in GIFT strain of Tilapia reared in recirculatory aquaculture system for 56 days^22^ and tanks for 30 days^62^; in Channel catfish, *Ictalurus punctatus* cultured in recirculating aquaculture system for 60 days^53^; in marbled eel, *Anguilla marmorata* cultured in recirculating aquaculture system for 71 days^63^; in Amur sturgeon, *A. schrenckii* cultured in concrete ponds for 50 days^64^, in Nile tilapia, *O. niloticus* cultured in glass aquaria for 10 weeks^65^ and rohu, *L. rohita* transported for 36 h^66^ and 2 h 30 min in polythene bags^67^. That means any kind of unpleasant living condition or stress situation could lead to the injury of the hepatic function.

Total protein, albumin and globulin content in the serum plays a pivotal role in fish innate immune response; chiefly during stressful conditions such as dietary irregularity, high stocking density, infections due to disease and other environmental stress factors^68^. These parameters are also used as an indicator of humoral defence system^69^. The drop in serum protein, albumin and globulin and their ratio in the higher stocking density (HSD) was a clear image of compromised innate immunity which may be due to inhibition of protein synthesis, liver cell lesions, kidney dysfunction or malnutrition. The crowding stress has also badly affected the serum protein and albumin concentration in Nile tilapia, *O. niloticus* cultured in cages for four months^32^ and common carp, *C. carpio* cultured in biofloc system for 49 days^26^.

The neuroendocrine system controls the acute and chronic stress responses which release catecholamines and cortisol^70^, both the steroid are responsible for controlling the ionic regulation in the fish body by the ion concentrations and their exchange between the body and surrounding environment called osmoregulation^71^. The responses of fish to stress are related to the neuroendocrine system, which is a critical part of osmoregulatory adaptations^72^. The present study showed that the Na^+^ level was highest in LSD and it varied significantly (*P* < 0.05) between LSD and MSD; while the lowest levels of Cl^-^ and K^+^ was found in HSD and varied significantly (*p* < 0.05) between LSD and HSD. The result may be attributed to excessive blood flow in gills and the permeability of the epithelium, resulting in ionic losses in freshwater fish^73^. A similar finding was recorded in marine fish (Atlantic salmon, *Salmo salar*) cultured in the plastic tank for 14 days^74^ and freshwater fish (dourado *Salminus brasiliensis*) cultured in tanks for 80 days^10^; however contrasting results have been seen in the case of salmon smolts, *Salmo salar* reared in fibreglass tanks for 100 days^75^ and in rainbow trout, *Oncorhynchus mykiss* reared in raceways for 24 h^76^.

The analysis of digestive enzyme activity is pertinent since it indicates digestion, absorption and nutrient utilization capacity of fish^77^. The feed intake stimulates digestive enzyme secretions to break down nutrient particles into simpler building blocks^77^ (Dong et al. 2018). Insufficient secretion of the digestive enzyme leads to deficiency of nutrients as well as growth retardation^63,78^. The digestive enzymes were affected by the increase in stocking density since the crowding stress could force the fish body metabolism to channelize their energy towards coping with stress conditions. Thus, it can also imply that the digestion and utilization of feed in fishes could be affected by crowding stress caused by higher stocking densities^26^. In the present study, the amylase activity was decreased significantly with increasing stocking density. Crowding stress also caused the decline in amylase activity in olive barb, *P. sarana* cultured in cages for 90 days^7^; in common carp, *C. Carpio* cultured in biofloc for 49 days^26^; in rainbow trout, *O. Mykiss* cultured in tanks for 84 days^79^; in marbled eel, *A. marmorata* cultured in recirculating aquaculture system for 71 days^63^ and turbot, *Scophthalmus maximus* cultured in recirculating aquaculture system for 70days^80^. The increase in lipase activity with an increase in stocking density among the treatments with *L. rohita* indicates that the utilization of body fat during crowing stress led to increasing in lipase activity. In the present study, due to crowding stress, protease activity in fishes were found to decline with the increase in stocking density, although the change was not significant among LSD and MSD. The decrease in protease activity along with an increase in stocking density resounded as a sign of growth retardation which can also be proved by declining PER value. A similar correlation between stocking density, PER and protease activity has been proven in Asian seabass cultured in recirculating aquaculture system (RAS) for 60 days^81^. Earlier studies on Japanese flounder juveniles cultured for 39 days in tanks^82^ and on blunt snout bream cultured for 42 days in concrete ponds^78^ have agreed with the fact that higher stocking densities possibly leads to goblet cell damage in the gut and successive reduction in metabolic activities, which might also be the reason for growth retardation in the present study.

Energy metabolism in fish bodies can also be indicated by flesh composition^83^. In the present study, deterioration in growth parameters suggests a decline in metabolism. Several factors can be a reflection to change in the body flesh composition of the stocked fishes, including water quality, crowding stress, nutrient availability, feed intake and follow up utilization^33^. In the current study, a significant decline of the fat extract was noticed in HSD which can be attributed due to chronic crowd stressing in cages followed by lesser uptake of feed resulting in lower accumulation of lipid in fish fleshes. On other hand, crowding stress enhanced the process of lipid metabolism to meet the growing energy demand followed by decreased fat content in some other fish species such as Amur sturgeon, *A. Schrenckii* cultured in flow-through tanks for 60 days^84^; in juvenile blunt snout bream. *M. amblycephala* cultured in tanks for 12 weeks^24^ and Nile tilapia, *O. niloticus* cultured in glass aquarium for 12 weeks^85^. In contrast, fat content increased with increasing stocking density in African catfish, *Clarias gariepinus* cultured in traditional ponds for 70 days^86^ and rainbow trout, *O. mykiss* cultured in tanks for 75 days^87^. The dissimilar results of these above studies could be due to the differences in fish species, age, size and the exposure time of crowding stress. The crude protein content of fish muscle was increased significantly in HSD in comparison to LSD. The present result corroborates with an earlier study on *A. marmorata* cultured in recirculating aquaculture tanks for 71 days, where crude protein content increases (*p* > 0.05) with an increment in stocking density^63^.

The muscle pH is an important flesh quality parameter and under stressful conditions, fishes produce low pH muscle^11,88,89^. In the present study, lower pH was exhibited in higher densities. It is possibly induced by chronic crowding stress which stimulates lactate acid formation in muscle^53,89^. Similar findings of lower pH and water holding capacity were obtained in the case of rainbow trout, *O. mykiss* cultured in tanks for 75 days^87^. The DL and FLR in HSD displayed a significant increasing trend, which indicates the deterioration of flesh quality attributed to lower muscular pH. Previous studies show that DL and FLR are inversely correlated to muscular pH of channel catfish, *I. punctatus* cultured in recirculating aquaculture system for 60 days^53^.

RNA, as a reflection of protein synthesis and expected to increase with an increase in the growth of somatic tissues^90^, whereas DNA, which is a genetic carrier molecule, and the quantity remains constant^91^. The RNA: DNA is sensitive to nutrition and as an index of the cell’s metabolic intensity and closely related to protein synthesis^92^. In the present experiment, the concentration of DNA did not vary (*p* > 0.05) among the three stocking densities of *L. rohita* however the RNA and RNA to DNA ratio was significantly decreased (*p* < 0.05) with an increase in stocking densities. The present study was endorsed by previous studies in Japanese flounder, *Paralichthys olivaceus* reared in tanks for 43 days^93^; in rohu, *L. rohita* cultured in fibreglass aquaria for 90 days^94^ and catla, *C. catla* cultured in polyvinyl circular troughs for 12 weeks^95^.

In the present study, Pearson correlation coefficient and PCA analysis depicted growth attributes like FBW, WG, SGR, AGR, survival, PER and FCE and biochemical parameters namely serum protein, albumin, globulin, T3, T4, and IGF1 are strongly correlated. However, a negative correlation was established among serum glucose, cortisol, SGOT, SGPT, SOD and CAT. These correlated parameters are interdependent to each other and their responses are similar under crowding stress. A similar observation was reported in *P. sarana* reared in cages for 90 days^7^. The outcome of PCA analysis revealed that stress responses negatively influenced while, decreased in the level of digestive enzymes (amylase and protease), serum protein and thyroid hormones (T3, T4 and IGF1) positively influence the growth attributes. Cluster analysis depicted a separate cluster by HSD indicates the bio-markers significantly differ in HSD comparison to LSD and MSD.

## Material and methods

### Ethical statement

The study protocol and the experiment conducted was approved by the ethical committee of ICAR-Central Inland Fisheries Research Institute, Barrackpore (IAEC/2020/04). All methods were carried out in accordance with relevant national and international guidelines and regulations. The study is in compliance with the Animal Research: Reporting of In Vivo Experiments (ARRIVE) guidelines (https://arriveguidelines.org/arrive-guidelines/experimental-procedures).

### Experimental facilities and animals

The present study was carried out in Salia reservoir (N19° 48.3887ʹ and E85° 03.6983ʹ), Odisha, India (Fig. 3a and b) in floating rectangular cages made of high density polyethylene (HDPE) of dimension 6 m × 4 m × 4 m with an effective volume of 84 m^−3^ for a period of 240 days. Advance fingerlings of *L. rohita* (14.25 ± 0.19 cm and 30.35 ± 1.08 g) were stocked at three different stocking densities, namely LSD (10 m^−3^), MSD (20 m^−3^), and HSD (30 m^−3^) in triplicates as per complete randomized design (CRD). Fishes were fed with CIFRI CAGEGROW®, a commercial extruded floating feed (28% protein and 4% fat) at a rate of 3% of body weight twice (09.00 h and 16.00 h). The feed rations were adjusted based on the estimated fish biomass every week. During the study, antibiotics, chemicals and medicines were strictly avoided. The health status of fish was monitored on a regular basis (Fig. 3c).

### Growth performance and feed efficiency

The growth performances were assessed every week by randomly lifting 30 fishes from cages to adjust the feed ration however other parameters were studied at the end of the experiment. Prior to sampling, the feeding was withheld for 24 h. The parameters such as weight gain (WG), absolute growth rate (AGR), specific growth rate (SGR), total yield and survival were calculated as follows: weight gain (g) = FBW (g)–IBW (g) where IBW = Initial body weight (g), FBW = Final body weight (g), absolute growth rate = [FBW (g)–IBW (g)]/culture period (days), specific growth rate (%) = (In FBW–In IBW)  × 100/culture period (days), yield (kgm^−3^) = total biomass of fish harvested per m^3^ volume at the end of experiment, survival (%) = (number of fish harvested/number of fish stocked) × 100. The feed efficiency was assessed by feed conversion ratio (FCR), feed conversion efficiency (FCE) and protein efficiency ratio (PER) and were calculated as follows: FCR = dry weight of diet fed (g)/wet weight gain of fish (g), FCE = wet weight gain of fish (g)/dry weight of diet fed (g), PER = wet weight gain of fish (g)/crude protein intake (g).

### Serum sample collection and preservation

Five fish from each cage were randomly collected and anaesthetized using clove oil @ 50 µL per litre water. The blood sample was collected using 2 mL sterile disposable syringe, by puncturing the caudal vein and kept in 1.5 mL eppendorf tube for 30 min for coagulation. The blood samples were centrifuged @ 4000 rpm for 10 min at 4 °C, the straw-coloured serum was pipetted out. The serum samples were carried to the laboratory in dry ice and stored at − 80 °C for further analysis.

### Serum biochemical indices

The automated blood biochemistry analyzer (Transaia-Erba, EM-2000, USA) was used to measure serum parameters such as protein, albumin, glucose, serum glutamate oxaloacetate transaminase (SGOT) and serum glutamate pyruvate transaminase (SGPT) using necessary reagents provided by the manufacturer. With the additional use of the ISE module in the same instrument, the electrolytic balance of fish such as Na^+^, K^+^ and Cl^−^ were estimated. Serum globulin was calculated by subtracting serum albumin from serum protein, and the A/G ratio was calculated as albumin/globulin.

Superoxide dismutase (SOD) was assayed using carbonate-bicarbonate buffer (0.1 M) with epinephrine (3 mM)^96^. The change in optical density was measured at 480 nm (BioTek’s Epoch™ 2). Catalase (CAT) activity was analyzed using phosphate buffer (50 mM, pH 7.0) with H_2_O_2_ solution^97^. The change in OD value was measured at 240 nm (BioTek’s Epoch™ 2).

Cortisol, tri-iodothyronine (T3), thyroxine (T4) and insulin like growth factor 1 (IGF1) in serum were quantified using an enzyme-linked immune survey assay (ELISA) kit (BT BioAssay, Shanghai, China) as per the manufacturer’s instruction and the final OD value was taken at 450 nm (BioTek’s Epoch™ 2).

### Digestive enzyme assay

The fish were dissected from each treatment and the gut tissues were kept in 0.25 M sucrose solution. The samples were homogenized using tissuelyser (Qiagen, Hilden, Germany), and centrifuged at 10,000 rpm for 10 min in 4 °C and the supernatant was collected to store in − 80 °C for further analysis. Amylase activity was assayed using 3,5-dinitrosalicylic acid method^98^ by observing reducing sugar production by the glucoamylase and α-amylase. Lipase activity was measured using phenolphthalein indicator based titration method^99^. The casein digestion method (triphosphate buffer- pH 7.8, trichloroacetic acid) was performed to measure protease activity^100^.

### Chemical composition of muscle and flesh quality

Fish flesh samples (n = 3) from all the treatments were collected. The proximate composition was assessed as per^101^. Moisture content was estimated via desiccation in an oven at 105 °C for 30 min and then cooling and weighing to a constant weight. Crude protein (nitrogen × 6.25) was determined by the Kjeldahl method using the kjeltec System (Tecater 1002 Distilling Unit), crude fat content was evaluated by using extraction with ether by soxtech system (Tecater 1043 Extraction Unit). The ash content was assessed by incineration in a muffle furnace at 550 ± 10 °C for 12 h. Muscular pH (*n* = 6) was measured in three different parts of the body (two dorsal sides and one caudal side), then the average was calculated. The fish was dissected and inserted a pH probe (Thermo Orion A211) into the muscular part. Before every use, the probe was calibrated with pH 4 & pH 7 buffer. Drip loss (DL) was calculated by weighing and keeping the sample in a vacuum polythene bag and storing it at 4℃ for 72 hr^102^. After thawing, the fish fillets were taken out from the bag and wiped with a paper towel and weighed. The calculation of the drip loss was based on the difference of initial sample weight and the weight after 72 h. DL = {100 × (Initial weight-weight after 72 h)/Initial weight}. For FLR, the sample was weighed and kept in a vacuum polythene bag and stored at − 20 °C for 72 h^103^. After thawing, the fish fillets were taken out from the bag and wiped with a paper towel and weighed. The calculation of FLR was as follows; FLR = [100*(Initial weight − weight after 72 h)/Initial weight].

### Nucleotide ratio

Fish muscle tissue was collected in RNA*later* and 70% ethanol for RNA and DNA isolation. DNA and RNA from muscle tissue were extracted using DNeasy and RNeasy kit (Qiagen Hilden, Germany) respectively as per manufacturer instruction. The concentration of DNA and RNA samples were measured by Nanodrop plate at 260/280 (BioTek’s Epoch™ 2, USA). After quantification, the RNA/DNA ratio was calculated.

### Water quality parameters

With the help of an advanced multiparameter probe (YSI PRO-DSS) and Aquaread probe (AP-7000), the water quality parameters such as temperature, dissolved oxygen (DO), pH and specific conductivity were analysed onsite. The standard protocols were followed to estimate water quality parameters like alkalinity, hardness, total nitrogen and total phosphorus^104^. The Secchi disc was used to measure water transparency.

### Statistical analyses

The data generated from all the parameters were subjected to one-way analysis of variance (ANOVA) and Duncan’s multiple range test to determine significant differences among the means and undergone principal component analysis, using statistical software SPSS 22.0, in which *P* < 0.05 considered statistically significant. Pearson correlation and hierarchical clustering analysis were carried out using PAST, 4.03 software.

## Conclusion

In conclusion, the present study accentuates that stocking density has a distinctive effect on fish physio-biochemical responses and the importance of evaluating density stress, towards determining optimal density to warrant the fish production. The stocking density mediated crowding stress negatively affects the growth attributes like WG, SGR and percentage survival. Poor digestive enzymes (amylase and protease) activity and flesh quality, fall of thyroid activity (T3, T4 and IGF1) and serum electrolytes imbalance has been perceived with increment in stocking density. However, elevated levels of the stress response like SOD, CAT, SGOT, SGPT, serum cortisol and glucose were encountered at higher stocking density. Based on growth attributes and multiple biomarker responses it is suggested that the optimum stocking density of Indian major carp (*L. rohita*) for tropical inland open water cage culture is 10 m^−3^. This is the first-ever attempt in inland cage reared fish to optimize stocking density based on multiple biomarker responses.
